# Supplementary material for: Dietary Patterns, Not Gut Microbiome Composition, Are Associated with Behavioral Challenges in Children with Autism: An Observational Study
Source: Nutrients. 2025 Nov 4;17(21):3476. doi: 10.3390/nu17213476 (PMC12610858; doi:10.3390/nu17213476)
Supplement: Supplementary file 1 [file nutrients-17-03476-s001.zip › Supplementary Table S2.pdf]

Table S2. STORMS (Strengthening the Organization and Reporting of Microbiome Studies) Checklist.

Section	Item	Description	How Addressed in This Study
**Title and Abstract**	1	Indicate study type, microbiome data type, and main exposure/outcome	The title and abstract specify a cross-sectional family-based study investigating gut bacterial and fungal microbiota (16S rRNA and ITS) and dietary patterns in ASD.
**Background and Rationale**	2	Provide scientific background and rationale	Introduction describes ASD epidemiology, gut–brain axis relevance, diet–microbiome hypotheses, and knowledge gaps motivating the study.
**Objectives**	3	Clearly state study objectives/hypotheses	The study aimed to characterize microbial and dietary differences among ASD children, siblings, and parents, and explore diet–microbiome associations.
**Study Design**	4	Describe study design and recruitment	Cross-sectional, family-based design including ASD children, non-ASD siblings, and parents. Recruitment described in Methods.
**Study Population**	5	Eligibility criteria and recruitment details	Inclusion/exclusion criteria, ASD diagnosis confirmation, and family recruitment described in Methods.
**Sample Size**	6	Report sample size and rationale	14 families (n = 53) for 16S and ITS analysis. 21 families (n = 79) for diet analysis. A sample size limited by sequencing cost; noted as a limitation.
**Ethical Approval**	7	State ethics approval and consent procedures	Approved by institutional ethics committee; informed consent obtained from all participants or guardians.
**Sample Collection**	8	Describe sample type, collection, and storage	Stool samples collected at home, immediately frozen, and stored at −80 °C until DNA extraction.
**Laboratory Methods**	9	DNA extraction and sequencing details	DNA extracted using standard kits; bacterial 16S rRNA (V3–V4) and fungal ITS1–ITS4 regions sequenced on Ion Torrent S5.
**Bioinformatics Processing**	10	Describe pipelines, databases, and quality filtering	Raw reads processed via CosmosID-QIIME2 integration; 97 % OTU clustering; SILVA v138 for taxonomy; quality thresholds and rarefaction described in Methods.
**Data Normalization**	11	Describe normalization and transformation	Counts converted to relative abundances; samples rarefied to 10,000 reads for diversity analyses.

**Diversity Analyses**	12	Define metrics used	α-diversity (Shannon, Simpson) and β-diversity (Bray–Curtis, weighted UniFrac) computed.
**Statistical Analyses**	13	Describe statistical tests and corrections	Kruskal–Wallis, Wilcoxon, and Linear Mixed-Effects Models (LMM) accounting for family clustering. PERMANOVA (adonis2) for β-diversity. Multiple testing corrected via Benjamini–Hochberg FDR.
**Confounders**	14	Identify and adjust for confounders	Age and sex included in models; family considered a random effect in LMM.
**Quality Control**	15	Report read depth, filtering thresholds, and contamination controls	Sequencing depth thresholds (>10,000 reads), chimera removal (DADA2), and low-abundance ASV exclusion described. Negative controls noted in Methods.
**Results Summary**	16	Summarize α- and β-diversity, and taxonomic differences	Presented in Results section with figures and PERMANOVA tables (Tables 3–4).
**Dietary Data**	17	Describe dietary assessment method	7-day food diaries analyzed by nutrient category; limitations discussed (recall bias, lack of nutrient-level quantification).
**Interpretation and Limitations**	18	Discuss biological interpretation and limitations	Discussion addresses sensory sensitivities, diet–microbiome pathways, lack of SCFA/inflammatory biomarkers, and sample size limitations.
**Data Availability**	19	Provide access details for data and code	Sequencing data available upon request; analysis performed in R using documented pipelines.
**Reproducibility**	20	Provide reproducible workflow or repository	R scripts for LMM and PERMANOVA provided upon request.
**Funding and Conflicts**	21	Report funding and conflicts of interest	Funding and conflict statements provided in the Acknowledgments section.
